# Aesthetic Surgery Training during Residency in the United States: A Comparison of the Integrated, Combined, and Independent Training Models

**DOI:** 10.1155/2014/281923

**Published:** 2014-08-24

**Authors:** Arash Momeni, Rebecca Y. Kim, Derrick C. Wan, Ali Izadpanah, Gordon K. Lee

**Affiliations:** ^1^Division of Plastic and Reconstructive Surgery, Stanford University Medical Center, 770 Welch Road, Suite 700, Palo Alto, CA 94305-5715, USA; ^2^Division of General Surgery, Stanford University Medical Center, Stanford, CA 94305, USA; ^3^Division of Plastic and Reconstructive Surgery, McGill University Health Centre, Montreal, Canada

## Abstract

*Background*. Three educational models for plastic surgery training exist in the United States, the integrated, combined, and independent model. The present study is a comparative analysis of aesthetic surgery training, to assess whether one model is particularly suitable to provide for high-quality training in aesthetic surgery. *Methods*. An 18-item online survey was developed to assess residents' perceptions regarding the quality of training in aesthetic surgery in the US. The survey had three distinct sections: demographic information, current state of aesthetic surgery training, and residents' perception regarding the quality of aesthetic surgery training. *Results*. A total of 86 senior plastic surgery residents completed the survey. Twenty-three, 24, and 39 residents were in integrated, combined, and independent residency programs, respectively. No statistically significant differences were seen with respect to number of aesthetic surgery procedures performed, additional training received in minimal-invasive cosmetic procedures, median level of confidence with index cosmetic surgery procedures, or perceived quality of aesthetic surgery training. Facial aesthetic procedures were felt to be the most challenging procedures. Exposure to minimally invasive aesthetic procedures was limited. *Conclusion*. While the educational experience in aesthetic surgery appears to be similar, weaknesses still exist with respect to training in minimally invasive/nonsurgical aesthetic procedures.

## 1. Introduction

The American Board of Plastic Surgery currently approves two educational models for plastic surgery, the integrated and independent model [1]. As defined by the Board, residents in the former complete all training within the same training program, whereas residents in the independent model complete the prerequisite training outside of the plastic surgery residency process. A third model, the combined or coordinated model, represents a program in which residents complete the prerequisite general surgery training in the same institution as, but not within, the plastic surgery program [1]. While the integrated model was initially regarded as an experimental model of surgical training, the challenges of an ever increasing complexity of plastic surgery have resulted in acknowledgment that more time should be spent in plastic surgery, thus resulting in an increasing popularity of the integrated model [2–4].

Advances in plastic surgery are seen not only in reconstructive but also in aesthetic surgery. In particular, minimally invasive/nonsurgical aesthetic procedures are in high demand, with a 231 percent increase from 1997 to 2009 [5]. Over 10 million minimally invasive aesthetic surgery procedures were performed in 2011 alone [6]. The challenges of mastering the expanding scope of aesthetic surgery upon graduation have resulted in an appreciation amongst leaders in the field that more emphasis should be placed on aesthetic surgery during residency training [7]. Despite the intuitive superiority of the integrated model, debate exists as to the ideal model for training of future plastic surgeons.

Previous studies by Morrison et al. and Oni et al shed light on the status of aesthetic surgery training among plastic surgery residents in the United States [5, 8]. However, no prior study has performed a head-to-head comparison of the training experience within the three existing plastic surgery training models. The present study represents a comparative analysis of aesthetic surgery training within these three training models to assess whether one model is superior in providing high-quality training in aesthetic surgery.

## 2. Methods

An online survey (SurveyMonkey, Menlo Park, CA) was developed to assess the quality of training in aesthetic surgery among plastic surgery residents in the United States. Content validity of the survey was determined by group consensus of the authors. Every question was critically assessed to minimize misinterpretation and ambiguity [9].

The 18-item survey had three distinct sections:

1. demographic information (postgraduate year- (PGY-) level, type of training program (integrated, combined, and independent), and gender);
2. current status of aesthetic surgery training, such as presence of a dedicated aesthetic surgery rotation, length of aesthetic surgery rotation (when offered), availability of resident aesthetic surgery clinics, number of aesthetic surgery procedures performed as primary surgeon, and predicted number of cosmetic surgery cases performed by the end of residency training;
3. respondents' perception regarding the quality of aesthetic surgery training, such as areas in which further time should be spent to improve skills, level of confidence with different procedures, minimum number of cases that need to be performed to feel confident/competent, and need for additional training in aesthetic surgery (fellowship).

The survey focused on the following procedures: breast augmentation, rhinoplasty, blepharoplasty, liposuction, abdominoplasty, facelift, mastopexy, brachioplasty, and breast reduction [9, 10]. An additional focus was assessing the status of clinical training in the most commonly performed minimally invasive/nonsurgical cosmetic procedures. These included skin care, botulinum toxin A, soft-tissue fillers, chemical peel, and laser treatment. Data were collected via multiple-choice questions, Likert scale selections, “yes-no” answers, fill-in-the-blank questions, and areas for open-ended written comments [9].

Residency program coordinators were contacted in March 2011 and were asked to assist in distributing the survey via email. Data collection was completed in June 2011. Only completed surveys were included in the final analysis. Only plastic surgery residents were contacted, whereas program directors were intentionally not included in the study as it has been demonstrated that significant differences exist among these two groups with respect to the perceived quality of training, with a trend to overestimate the quality of training by program directors [8]. As such, we were mostly interested in the perceptions and opinions of plastic surgery residents.

Only the responses of senior residents (PGY-4 and above) were used for final analysis. A comparative analysis of residents in integrated, combined, and independent programs was performed.

## 3. Statistical Analysis

Final data analysis was performed in STATA 9.0 (STATA Corporation, College Station, TX, 2006). Exploratory analyses of continuous data included histograms, means, and standard deviations for normally distributed data and medians and interquartile ranges for nonnormally distributed data. Normality of the continuous variables was confirmed with the Shapiro-Wilk test using a critical *P* value of 0.05. For categorical data, tables were generated showing frequencies and percentages.

Bivariate analyses were conducted to assess the association between two variables. For the comparison of categorical versus continuous variables, Kruskal-Wallis test measured for differences in medians. *n*-by-*n* tables were generated to test for associations between categorical variables with the chi-squared test or Fisher's exact test.

## 4. Results

A total of 86 senior plastic surgery residents completed the survey. Of the respondents, 76.7 percent were male (*N* = 66) and 23.2 percent were female (*N* = 20). Twenty-three, 24, and 39 residents were in integrated, combined, and independent residency programs, respectively. The proportion of female residents was 26.1 percent (*N* = 6), 33.3 percent (*N* = 8), and 15.4 percent (*N* = 6) in integrated, combined, and independent programs, respectively (*P* = 0.24).

The majority of residents in integrated (*N* = 15; 65.2 percent) and independent (*N* = 29; 59 percent) programs reported having a dedicated aesthetic surgery rotation in contrast to 45.8 percent (*N* = 11) of residents in combined programs (*P* = 0.38). The median length of this rotation (when offered) was 4 months, 3 months, and 3 months in integrated, combined, and independent programs, respectively (*P* = 0.07). The majority of residents in integrated (*N* = 16; 69.6 percent), combined (*N* = 20; 83.3 percent), and independent (*N* = 32; 82.1 percent) programs reported that formal training in aesthetic surgery was incorporated in other rotations as well (*P* = 0.44). A statistically significant difference was seen with respect to the presence of resident cosmetic surgery clinics. Almost half of all residents in integrated (*N* = 11; 47.8 percent) and independent (*N* = 19; 48.7 percent) programs reported to have such a training opportunity in contrast to the majority of residents in combined programs (*N* = 20; 83.3 percent) (*P* = 0.01).

Although the majority of plastic surgery residents in combined programs (*N* = 15; 62.5 percent) reported to have performed more than 20 aesthetic surgery cases as the primary surgeon at the time of the survey compared to 34.8 percent and 41 percent for the integrated and independent programs, respectively (*P* = 0.21), the predicted number of aesthetic surgery cases performed upon completion of training was fairly similar among the three training models (*P* = 0.98) (Table 1).

Given the rise of minimally invasive/nonsurgical cosmetic procedures [5, 8], one focus of the present study was to assess how well senior residents were trained in these areas and whether differences existed among the various training models. No statistically significant differences were observed within the categories analyzed. The majority of residents in integrated, combined, and independent programs indicated to have additional training in administration of botulinum toxin A and use of fillers (Table 2).

As an indirect marker of quality of training, senior residents were asked to rate their level of confidence with each procedure using a Likert scale (1: not confident, 5: very confident). The highest median values were achieved for breast reduction and abdominoplasty with lowest values for rhinoplasty (Table 3). These findings were furthermore confirmed after subgroup analysis of residents who reported to be “confident” and “very confident” with the respective procedures (Table 4). Of note, the only statistically significant difference among the study groups was seen for “breast augmentation” with a significantly higher number of residents in combined programs reporting to be “confident” or “very confident” with this procedure (*P* = 0.05).

When offered the opportunity to spend more time to improve skills in any given area, “rhinoplasty” was the procedure chosen by the majority of senior residents (more than 87 percent). Interestingly, of the areas where additional training was desired 3 of the top 5 areas were nonsurgical, that is, skin care, chemical peels, and laser resurfacing. Senior residents in combined programs were significantly less likely to spend additional time in “breast augmentation” when compared to residents in integrated and independent programs (*P* = 0.03) (Table 5). This finding supports the previous finding; namely, residents in combined programs were significantly more likely to feel “confident” or “very confident” with this procedure.

Table 6 displays residents' opinion regarding the minimum number of aesthetic surgery cases required to achieve competency. No significant differences were seen among study groups. In almost all categories, residents felt that more than 8 cases as primary surgeon were necessary to provide sufficient training to allow competent execution of the procedure upon graduation.

When asked to rate the quality of aesthetic surgery training on a scale of 1 to 5 (1: poor, 5: excellent), a value of >4 was reported by 11 (47.8 percent), 15 (62.5 percent), and 20 (51.3 percent) residents in integrated, combined, and independent programs, respectively (*P* = 0.58). About a third of residents in each training model reported to have an interest in a predominantly aesthetic surgery practice (integrated: *N* = 7 (30.4 percent); combined: *N* = 7 (29.2 percent); independent: *N* = 12 (30.8 percent)) (*P* = 0.99). Similarly, a third of residents felt that additional training in aesthetic surgery (i.e., fellowship) was necessary (integrated: *N* = 8 (34.8 percent); combined: *N* = 8 (33.3 percent); independent: *N* = 12 (30.8 percent)) (*P* = 0.94).

## 5. Discussion

Advances in plastic surgery and the increasing complexity of the specialty have triggered discussions regarding the ideal training model in order to meet the challenges of providing adequate training in plastic and, in particular, aesthetic surgery [11, 12]. Consensus seems to exist that improving the quality of aesthetic surgery training during residency is critical [13]. Leaders in the field have commented that only through improvement in the quality of training will plastic surgery as a specialty continue to prosper [7]. Undoubtedly, without high-quality training, plastic surgeons will face difficulties distinguishing themselves from competing specialties. Specialties such as otolaryngology already have demonstrated great interest in incorporating aesthetic surgery into their specialty [14]. Studies from Brazil, Italy, Germany, England, and Canada emphasize the importance of comprehensive training in aesthetic surgery to prepare plastic surgeons for the demands and challenges ahead [9, 15–17].

The present study can be regarded as a follow-up study to the work by Morrison et al. and Oni et al. with the addition of a comparative analysis to assess whether differences exist among the three existing training models in the United States: integrated, combined, and independent models [5, 8]. This is in contrast to the previous studies which did not differentiate between the various existing training models [8].

Similar to previous studies, the majority of residents in this study were male, without any notable difference among the three training models. Further similarities include the length of dedicated aesthetic surgery rotations (when offered), with a typical duration of 3 to 4 months [5, 8, 9].

It is encouraging to notice that the majority of residents confirmed that formal training in aesthetic surgery was incorporated in other rotations as well. This certainly reflects the awareness that aesthetic surgery assumes a significantly greater role in plastic surgery training than in years past [18].

The educational value of resident clinics and the importance of hands-on experience have been discussed and demonstrated [19–21]. In fact, such clinics have been considered a “compulsory component of any training program” [22]. Similar to previous studies, our results indicate that 58.1 percent of all respondents had access to resident clinics [5, 8]; however, subgroup analysis revealed that this was significantly more likely to be the case for residents in combined programs. The observation that a similar experience was reported with respect to case numbers and confidence levels with aesthetic surgery procedures may be explained by the fact that residents in integrated and independent programs, in return, were more likely to have a dedicated aesthetic surgery rotation. Although some authors have argued that the combined training model combines weaknesses of the other two, this notion could not be supported by the findings of this study [4, 23].

Based on the results of the present study, exposure to techniques of minimally invasive/nonsurgical aesthetic procedures is still rather limited amongst all residents surveyed. This is, furthermore, evidenced by the fact that of the areas where additional training was desired 3 of the top 5 areas were nonsurgical (i.e., skin care, chemical peels, and laser resurfacing). Notable exceptions are techniques in administration of botulinum toxin A and use of fillers. As such, although Oni et al. report on “increasing levels of resident confidence…in nonsurgical procedures,” it appears that further improvement is warranted [5]. Interestingly, although minimally invasive/nonsurgical aesthetic procedures demonstrate the sector with the most rapid increase in demand, studies with focus on aesthetic surgery training frequently do not comment on this sector [15, 17, 20]. An increasing awareness and understanding that these techniques must be mastered by graduating plastic surgery residents is critical, as lack of familiarity with these techniques results in a substantial disadvantage after graduation.

More than half of all responders reported that they perceived their training in aesthetic surgery as either “very good” or “excellent.” No significant differences were seen between residents in the different training models analyzed. The present study does, however, represent the first comparative analysis of the various training models leading to board certification in the United States. No objective (presence of dedicated aesthetic surgery rotations, number of aesthetic surgery cases performed, etc.) or subjective (level of confidence with aesthetic surgery procedures, perceived need for additional training, etc.) differences were seen among residents in integrated, combined, or independent programs. One may conclude that, at least with respect to aesthetic surgery training, an equivalent training experience seems to be provided. Any interpretation beyond this, however, is unsubstantiated. The results of this study should by no means be interpreted as proof that the quality of plastic surgery residency training is similar for the various training models.

## 6. Conclusion

The importance of aesthetic surgery training during residency has been recognized. The educational experience in aesthetic surgery among residents in integrated, combined, and independent residency programs is similar. Weaknesses still exist mainly with respect to training in minimally invasive/nonsurgical aesthetic procedures.

## Figures and Tables

**Table 1:** Number of aesthetic surgery cases as primary surgeon at the time of the survey versus upon completion of training.

	None	1–10	11–15	16–20	>20	*P* value	None	1–10	11–15	16–20	>20	*P* value
Integrated (*N* = 23) (%)	1 (4.4)	9 (39.1)	3 (13.0)	2 (8.7)	8 (34.8)	0.21	0 (0.0)	1 (4.4)	2 (8.7)	1 (4.4)	19 (82.6)	0.98
Combined (*N* = 24) (%)	1 (4.2)	5 (20.8)	3 (12.5)	0 (0.0)	15 (62.5)		0 (0.0)	2 (8.3)	3 (12.5)	0 (0.0)	19 (79.2)	
Independent (*N* = 39) (%)	5 (12.8)	9 (23.1)	9 (23.1)	0 (0.0)	16 (41.0)		1 (2.6)	4 (10.3)	3 (7.7)	2 (5.1)	29 (74.4)	

	Number of aesthetic surgery cases as primary surgeon *at the time of the survey *						Number of aesthetic surgery cases as primary surgeon *upon completion of training *					

**Table 2:** Number of senior plastic surgery residents with additional training in minimally invasive/nonsurgical cosmetic procedures.

	Integrated (*n* = 23)	Combined (*n* = 24)	Independent (*n* = 39)	*P* value
Skin care (%)	9 (39.1)	4 (16.7)	8 (20.5)	0.16
Chemical peels (%)	7 (30.4)	5 (20.8)	7 (18.0)	0.57
Laser resurfacing (%)	10 (43.5)	7 (29.2)	16 (41.0)	0.54
Botox (%)	15 (65.2)	20 (83.3)	29 (74.4)	0.39
Fillers (%)	13 (56.5)	20 (83.3)	23 (59.0)	0.08

**Table 3:** Median level of confidence with various aesthetic surgery procedures (1: not confident, 5: very confident).

	Integrated (*n* = 23)	Combined (*n* = 24)	Independent (*n* = 39)	*P* value
Face lift (range)	3 (1–4)	3 (1–4)	2 (1–3)	0.39
Blepharoplasty (range)	3 (3-4)	4 (2.5–4.5)	3 (2–4)	0.76
Rhinoplasty (range)	2 (1-2)	2 (1–3)	2 (1–3)	0.19
Breast augmentation (range)	4 (3–5)	4.5 (4-5)	4 (2–5)	0.16
Breast reduction (range)	5 (4-5)	5 (4-5)	5 (4-5)	0.73
Mastopexy (range)	4 (3–5)	4 (3–5)	4 (3-4)	0.16
Abdominoplasty (range)	5 (4-5)	5 (4-5)	5 (4-5)	0.32
Brachioplasty (range)	4 (3-4)	3 (3-4)	3 (2–4)	0.44
Liposuction (range)	4 (4-5)	4 (4-5)	4 (3–5)	0.45

**Table 4:** Proportion of residents feeling “confident” or “very confident” with the procedures of interest.

	Integrated (*n* = 23)	Combined (*n* = 24)	Independent (*n* = 39)	*P* value
Face lift (%)	6 (26.1)	10 (41.7)	7 (18.0)	0.12
Blepharoplasty (%)	10 (43.5)	13 (54.2)	19 (48.7)	0.76
Rhinoplasty (%)	0 (0.0)	4 (16.7)	4 (10.3)	0.15
Breast augmentation (%)	14 (60.9)	20 (83.3)	21 (53.9)	0.05
Breast reduction (%)	20 (87.0)	21 (87.5)	30 (76.9)	0.54
Mastopexy (%)	14 (60.9)	17 (70.8)	21 (53.9)	0.41
Abdominoplasty (%)	19 (82.6)	22 (91.7)	33 (84.6)	0.73
Brachioplasty (%)	12 (52.2)	11 (45.8)	15 (38.5)	0.57
Liposuction (%)	18 (78.3)	19 (79.2)	26 (66.7)	0.52

**Table 5:** Areas in which senior plastic surgery residents would spend more time to improve their skills.

	Integrated (*n* = 23)	Combined (*n* = 24)	Independent (*n* = 39)	*P* value
Face lift (%)	**16 (69.6)**	**17 (70.8)**	**31 (79.5)**	0.62
Blepharoplasty (%)	9 (39.1)	9 (37.5)	13 (33.3)	0.89
Rhinoplasty (%)	**20 (87.0)**	**21 (87.5)**	**34 (87.2)**	1.00
Breast augmentation (%)	6 (26.1)	1 (4.2)	12 (30.8)	0.03
Breast reduction (%)	3 (13.0)	1 (4.2)	4 (10.3)	0.58
Mastopexy (%)	7 (30.4)	2 (8.3)	11 (28.2)	0.11
Abdominoplasty (%)	4 (17.4)	2 (8.3)	4 (10.3)	0.63
Brachioplasty (%)	7 (30.4)	6 (25.0)	13 (33.3)	0.79
Liposuction (%)	5 (21.7)	3 (12.5)	8 (20.5)	0.73
Skin care (%)	**11 (47.8)**	**13 (54.2)**	**23 (59.0)**	0.70
Chemical peels (%)	**12 (52.2)**	**15 (62.5)**	**27 (69.2)**	0.41
Laser resurfacing (%)	**10 (43.5)**	**13 (54.2)**	**23 (59.0)**	0.51
Botulinum toxin A (%)	6 (26.1)	8 (33.3)	15 (38.5)	0.65
Fillers (%)	9 (39.1)	12 (50.0)	18 (46.2)	0.75

**Table 6:** Residents' opinion regarding the minimum number of cosmetic surgery procedures required as primary surgeon during residency training to achieve competency.

			1–3	4–7	8–10	>10	*P* value
Face lift	Integrated (*N* = 23) (%)		0 (0.0%)	5 (21.7%)	8 (34.8%)	10 (43.5%)	0.48
	Combined (*N* = 24) (%)		0 (0.0%)	6 (25.0%)	6 (25.0%)	12 (50.0%)	
	Independent (*N* = 39) (%)		2 (5.1%)	7 (17.9%)	18 (46.2%)	12 (30.8%)	

Blepharoplasty	Integrated (*N* = 23) (%)		0 (0.0%)	7 (30.4%)	9 (39.1%)	7 (30.4%)	0.25
	Combined (*N* = 24) (%)		1 (4.2%)	7 (29.2%)	5 (20.8%)	11 (45.8%)	
	Independent (*N* = 39) (%)		2 (5.1%)	3 (7.7%)	17 (43.6%)	7 (17.9%)	

Rhinoplasty	Integrated (*N* = 23) (%)		0 (0.0%)	4 (17.4%)	3 (13.0%)	16 (69.6%)	0.58
	Combined (*N* = 24) (%)		0 (0.0%)	2 (8.3%)	6 (25.0%)	16 (66.7%)	
	Independent (*N* = 39) (%)		2 (5.1%)	3 (7.7%)	11 (28.2%)	23 (59.0%)	

Breast augmentation	Integrated (*N* = 23) (%)		0 (0.0%)	5 (21.7%)	8 (34.8%)	10 (43.5%)	0.28
	Combined (*N* = 24) (%)		2 (8.3%)	4 (16.7%)	9 (37.5%)	9 (37.5%)	
	Independent (*N* = 39) (%)		2 (5.1%)	12 (30.8%)	18 (46.2%)	7 (17.9%)	

Breast reduction	Integrated (*N* = 23) (%)		1 (4.3%)	6 (26.1%)	6 (26.1%)	10 (43.5%)	0.51
	Combined (*N* = 24) (%)		1 (4.2%)	3 (12.5%)	10 (41.7%)	10 (41.7%)	
	Independent (*N* = 39) (%)		0 (0.0%)	9 (23.1%)	17 (43.6%)	13 (33.3%)	

Mastopexy	Integrated (*N* = 23) (%)		1 (4.3%)	5 (21.7%)	8 (34.8%)	9 (39.1%)	0.46
	Combined (*N* = 24) (%)		1 (4.2%)	6 (25.0%)	5 (20.8%)	12 (50.0%)	
	Independent (*N* = 39) (%)		1 (2.6%)	12 (30.8%)	16 (41.0%)	10 (25.6%)	

Abdominoplasty	Integrated (*N* = 23) (%)		1 (4.3%)	11 (47.8%)	6 (26.1%)	5 (21.7%)	0.78
	Combined (*N* = 24) (%)		2 (8.3%)	8 (33.3%)	8 (33.3%)	6 (25.0%)	
	Independent (*N* = 39) (%)		3 (7.7%)	15 (38.5%)	16 (41.0%)	5 (12.8%)	

Brachioplasty	Integrated (*N* = 23) (%)		1 (4.3%)	10 (43.5%)	7 (30.4%)	5 (21.7%)	0.78
	Combined (*N* = 24) (%)		2 (8.3%)	7 (29.2%)	10 (41.7%)	5 (20.8%)	
	Independent (*N* = 39) (%)		5 (12.8%)	17 (43.6%)	12 (30.8%)	5 (12.8%)	

Liposuction	Integrated (*N* = 23) (%)		1 (4.3%)	12 (52.2%)	4 (17.4%)	6 (26.1%)	0.13
	Combined (*N* = 24) (%)		4 (16.7%)	6 (25.0%)	8 (33.3%)	6 (25.0%)	
	Independent (*N* = 39) (%)		3 (7.7%)	16 (41.0%)	16 (41.0%)	4 (10.3%)	
